# Evaluation of recoverable potential of deep coalbed methane in the Linxing Block, Eastern Margin of the Ordos Basin

**DOI:** 10.1038/s41598-024-59128-x

**Published:** 2024-04-22

**Authors:** Bo Chen, Song Li, Dazhen Tang, Yifan Pu, Guanghao Zhong

**Affiliations:** 1 School of Energy Resources, China University of Geosciences (Beijing), Beijing, 100083 China; 2 Coal Reservoir Laboratory of National Engineering Research Center of CBM Development & Utilization, Beijing, 100083 China

**Keywords:** Linxing Block, Resource conditions, Development conditions, Key geological parameters, Recoverable favorable areas, Coal, Natural gas

## Abstract

The deep coalbed methane (CBM) resources are widely developed in the Linxing Block. However, the evaluation of CBM geological areas suitable for CBM exploitation remains unexplored, hindering further development. This research optimizes the key geological parameters that influence the development of deep CBM from the perspectives of resource and development conditions. The evaluation system for deep CBM recoverability has been established, and the multi-fuzzy evaluation method has been used to perform the quantitative evaluation of recoverability. The results indicate that the resource conditions of No.8 + 9 coal seam are superior to those of No.4 + 5 coal seam. Favorable resource conditions are predominantly concentrated in the northeast and specific southern portions of the research area. Favorable development conditions for both coal seams are mostly concentrated in the northeastern area. Based on the classification standard of recoverable favorable areas, the Level II area is crucial for the development of No.4 + 5 coal seam. This area is primarily distributed in the northeast of the research area., Both Level I and Level II areas for the No. 8 + 9 coal seam are situated in the northeast. The Level III area is earmarked for deep CBM production and shows potential for exploration. Further analysis reveals that the resource conditions in the favorable area are generally superior to the development conditions. These areas are classified as Class A, including categories such as I-A, II-A, and III-A, indicating relatively complex reservoir transformation.

## Introduction

CBM is a clean resource that exists within the pores and fractures of coal seams^1,2^. It has found commercial success worldwide^3^. The Fourth Resource Assessment reports that China’s the CBM resources are 11.93 × 10^12^m^3^ at the depth of 1500–2000 m and 18.47 × 10^12^m^3^ at the depth of 2000–3000 m, indicating significant potential for deep CBM^4^. Significant developments have occurred in the exploration and development of deep CBM in regions such as Yanchuannan Block, Daning-Jixian Block^5,6^, Daniudi Block^7^, Baijiahai area^8^, and the Dacheng area^9^. Deep CBM production from vertical wells has exceeded 3 × 10^4^/d, and horizontal wells have yielded over 10 × 10^4^/d^10,11^. The Linxing Block is a typical deep CBM block with abundant resources. However, exploration and development of deep CBM in Linxing Block has been slow, and the geological selective areas for deep CBM development have not been evaluated, severely limiting further development.

Efficient exploration and development of CBM resources relies on careful selection of geological areas conducive to CBM development. This selection process depends not only on understanding the in-situ resource conditions of CBM but also on assessing transformation of the coal reservoir, which is a key factor influencing the efficiency of CBM extraction. The recoverability of CBM represents the comprehensive gas production potential, which is influenced by both the resource conditions of CBM and the transformation of coal reservoirs. Recent studies have demonstrated promising resource conditions for deep CBM^6,10^. However, deep coal reservoir densification is severe and the effective transformation of reservoirs is difficult^12^. This is an important factor restricting the efficient exploitation of deep CBM resources. Therefore, the precise identification of high-yield areas within CBM resource-rich areas poses a formidable challenge in the development of deep CBM.

There are numerous quantitative methods available to assess CBM recoverability, including the AHP, principal component analysis, fuzzy matter-element method, multi-level fuzzy evaluation method, numerical simulation method, and grey clustering correlation analysis, etc.,^13–20^. Each approach possesses distinct attributes and applicable contexts, and a single factor cannot evaluate the recoverable potential of deep CBM. The multi-level fuzzy evaluation method addresses this limitation by hierarchically organizing the decision system, constructing a multi-level structural model, and enabling multidimensional analysis. This approach effectively navigates the intricacies and uncertainties associated with CBM recoverability assessment. Its demonstrated reliability and practicality make it a suitable choice for comprehensively evaluating deep CBM recoverability^17^.

In this study, a comprehensive analysis of the resource and development conditions of No.4 + 5 and No.8 + 9 coal seams in the Linxing Block are conducted. Firstly, key parameters that affect deep CBM development are identified. Subsequently, an evaluation framework for deep CBM recoverability is formulated based on the 'one-vote veto' concept. Finally, the multi-level fuzzy evaluation approach has been used to quantitatively assess deep CBM recoverability within the research area. This evaluation provides a foundational reference for future deep CBM exploration and development.

## Geological setting

**Figure 1:**
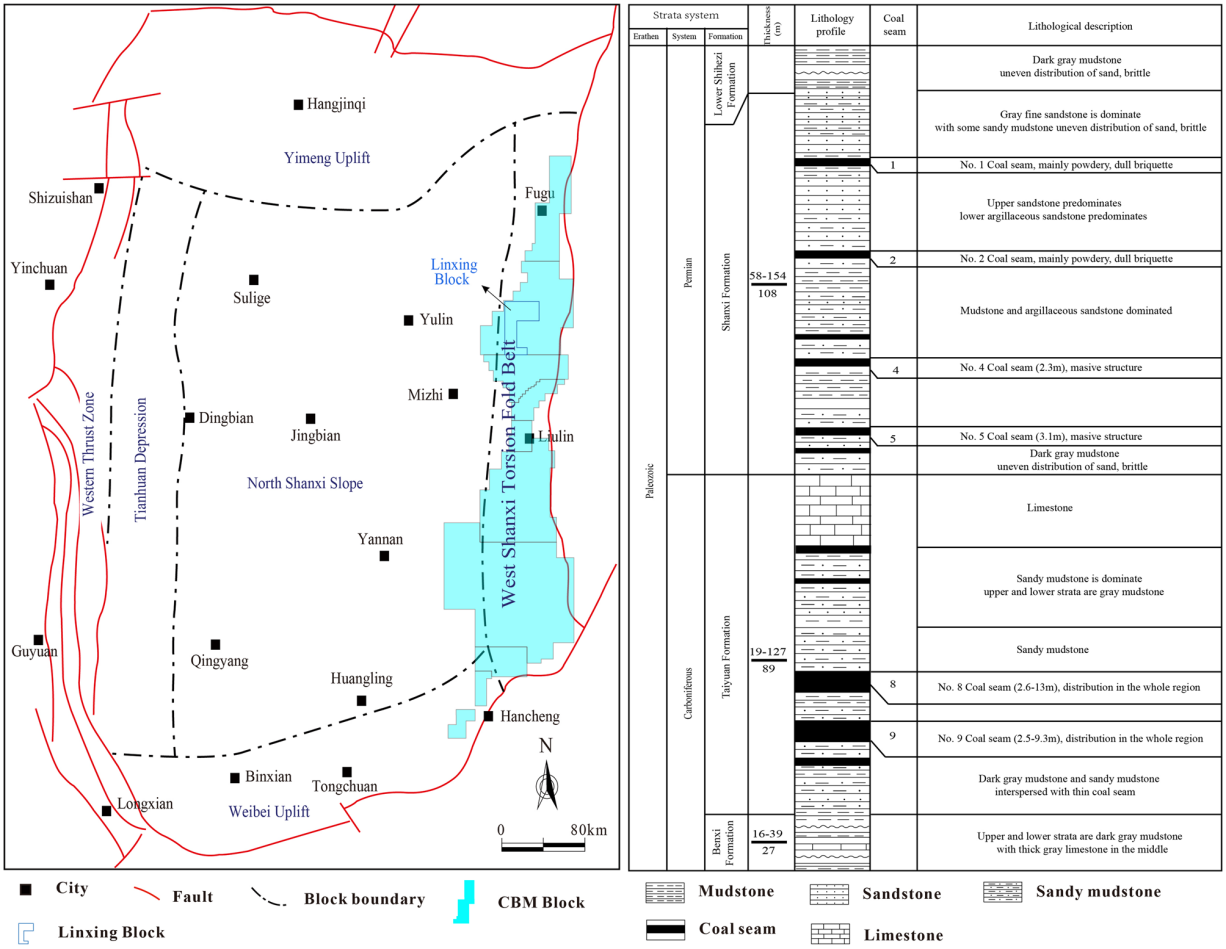
Geological characteristics of research area^21^.

The Ordos Basin is a large-scale superimposed basin that developed on the North China Craton. Its formation is related to the North China epicontinental basin, the Late Paleozoic sea-land interaction basin, and the Triassic and Jurassic super-large lake basins^21^. The structure in the basin is simple and stable, and its edge structure is active. The basin can be divided into six secondary tectonic units, including Yimeng uplift, Weibei uplift, North shanxi slope, Tianhuan depression, Western thrust zone, West shanxi torsion fold belt^21^. The West shanxi torsion fold belt is located at the eastern margin of Ordos Basin, which is the primary tectonic belt of CBM exploration and development (Fig. 1a).

The Linxing Block is located in the north-central part of the West shanxi torsion fold belt. The structure is simple, and the overall structure is NE -SW monoclinic structure. The central part of the research area exhibits an uplifted formation due to magmatic intrusions, accompanied by the radial development of faults^21–24^. The Paleozoic strata in the research area is developed (Fig. 1b), and a large amount of unconventional natural gas resources (CBM, shale gas and tight sandstone gas) have been discovered. The main strata for CBM exploitation are the Permian Shanxi formation and Carboniferous Taiyuan formation. The Taiyuan formation is formed in tidal-dominated delta environment and consists of limestone, sandstone, mudstone, sandy mudstone and several sets of coal seams. Among these, the No.8 + 9 coal seam is the main mineable coal seam. Shanxi formation is formed in a shallow-water delta environment and consists of sandstone, mudstone, sandy mudstone and several sets of coal seams. Among these, the No.4 + 5 coal seam is the main mineable coal seam. The two sets of coal seams consistently span the entire area, with burial depths exceeding 1600 m.

## Samples and methods

### Sampling and experiments

The study utilized experimental data provided by the China National Offshore Oil Corporation. Coal samples were collected from the Linxing Block. A total of ten coal samples were tested for vitrinite reflectance of coal according to People’s Republic of China standard GB/T 6948–2008. The coal samples were subjected to proximate analysis (according to the national standard GB/T 212–2001) to determine ash, moisture, volatile matter, and fixed carbon. The methane isothermal adsorption experiment was carried out in accordance with the People's Republic of China standard GB/ T 19,560–2004. The water quality analysis of coal seam water was performed following the China geological and mineral industry standards DZ/T 0064.49–1993, DZ/T 0064.51–1993, and the People's Republic of China standard GB/T 5750.6–2006. Additionally, the data used in the geological map such as structural features, coal seam thickness, burial depth, roof and floor lithology, were all obtained from field data and 38 CBM well exploration reports.

## Establishment of deep CBM evaluation system

### Optimization of key parameters for deep CBM evaluation

The exploitation of CBM is influenced by various factors, with increasing burial depth notably complicating the exploitation of deep CBM. Consequently, evaluating the recoverability of deep CBM should not only consider the resource conditions of deep CBM but also account for the development conditions that affect the transformation of deep coal reservoirs.

Zhou et al.,^10^ claimed that the material foundation for deep CBM in China is generally better when deep coal reservoirs are better preserved. Moreover, a direct correlation exists between higher free gas content and a shorter gas breakthrough time for CBM, resulting in swifter production ascent. Consequently, certain parameters stand out as crucial in characterizing deep CBM resource conditions, including coal seam thickness, gas saturation, gas content, resource abundance, hydrogeological characteristics, as well as lithology of coal roof and floor. The notably compact nature of deep coal reservoir presents a critical constraint to the efficient exploitation of deep CBM. As an important approach of deep CBM exploitation, reservoir fracturing transformation is controlled by the mechanical properties of coal seams and the external stress conditions at significant depths. The high in-situ stress conditions govern coal reservoir fracturing. Therefore, parameters such as permeability, elastic modulus, stress distribution, horizontal stress differential, and microstructural characteristics are selected to depict the development state of deep coal reservoirs in this research.

## Establishment of deep CBM recoverable evaluation system

**Figure 2:**
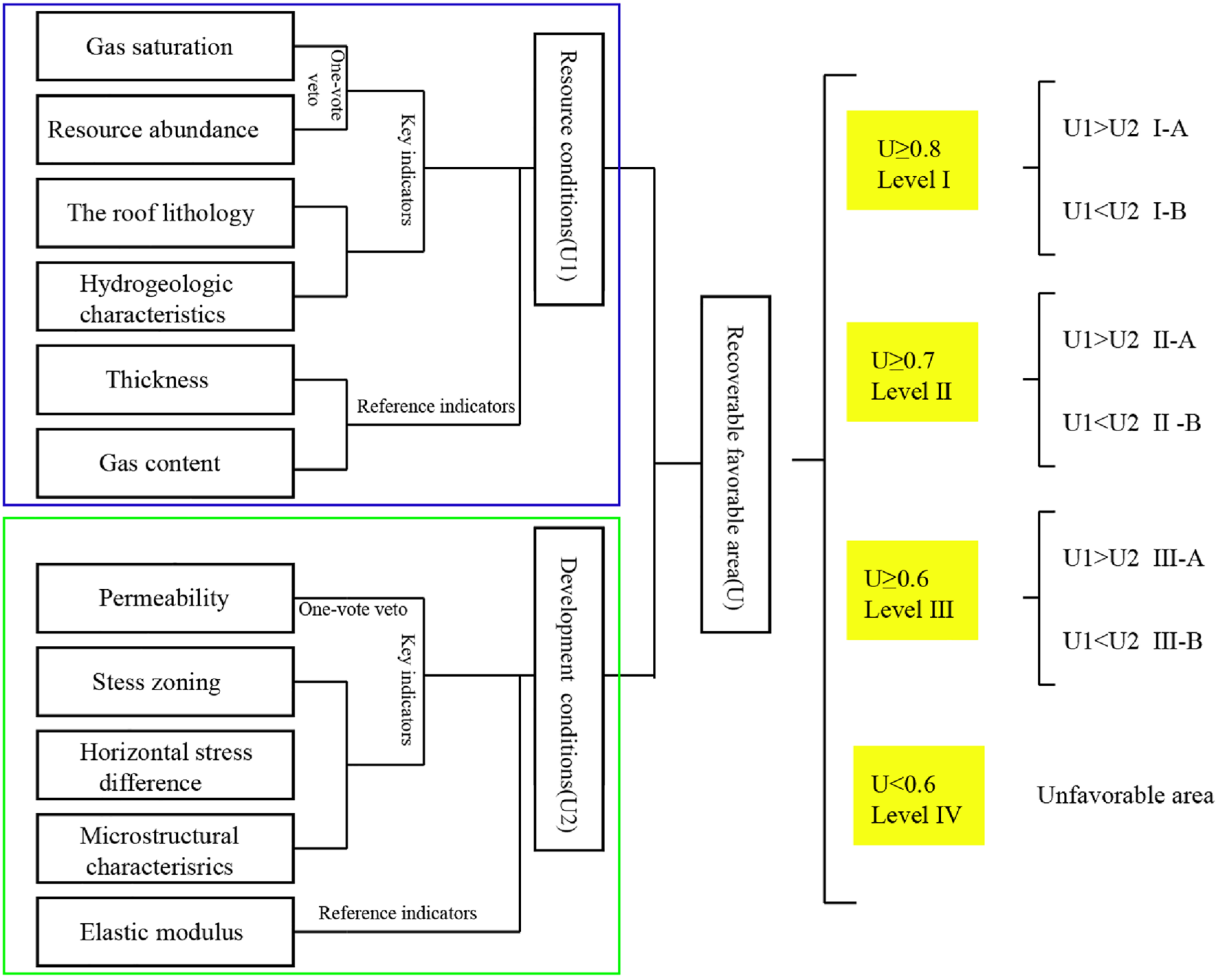
Evaluation system of deep CBM recoverability and the classification standard of recoverable favorable areas.

The evaluation of favorable CBM areas involves several key parameters, with certain factors holding decisive significance, akin to a one-vote veto principle. Regions with inadequate key parameters are excluded from the evaluation. Meanwhile, some parameters offer valuable reference points for CBM area evaluation. Grounded in this concept, the study has developed an evaluation system for deep CBM recoverability, accompanied by a classification standard for recoverable favorable areas within the study zone (Fig. 2). Yan et al.,^6^ claimed that high yields are not necessarily correlate with deep CBM enrichment, instead, it hinges on the effective degree of deep reservoir transformation. Therefore, further analysis of the recoverable favorable areas shows that when the resource conditions are better than the development conditions, it is classified as Class A, otherwise it is classified as Class B. This provides a clear reference for the later development of deep CBM, and different reservoir transformation techniques are used for different conditions to finally realize the efficient exploitation of deep CBM.

Deep CBM resource conditions can be evaluated using four key parameters: resource abundance, gas saturation, lithological conditions of coal roof and floor, and hydrogeological characteristics. It is important to note that higher gas saturation of coal seam enhances gas desorption and stable CBM well production. Similarly, increased resource abundance leads to extended CBM exploitation timelines and augmented commercial benefits. Therefore, resource abundance and gas saturation have the significance of one-vote veto. Additionally, gas content and thickness are reference indicators that interdependently define high resource abundance and eliminate single-factor-driven overestimations.

The pronounced depth of coal reservoir burial entails intricates in-situ stress conditions. Triaxial compression of coal seams triggers fracture closure, which reduces porosity and permeability, ultimately affecting CBM production negatively. Fracturing coal reservoir is complicated by elevated in-situ stress, and the effectiveness of transformation varies across microstructural zones. Evaluating the development conditions of deep coal reservoir involves pivotal indicators, including permeability, stress zoning, horizontal stress difference (HSD), and microstructural attributes. Permeability, being a paramount determinant of CBM productivity, plays a significant role as a one-vote veto parameter. Given that elastic modulus significantly influences hydraulic fracture expansion, it stands as a pertinent reference indicator.

## Quantitative evaluation modeling of deep CBM recoverable favorable area

The multi-level fuzzy evaluation method is a comprehensive evaluation method^16,17^ that combines qualitative and quantitative indicators of complex problems to achieve quantitative evaluation of complex problems. The details of general principles, mathematical processes, evaluation parameters and uncertainty of the multi-level fuzzy evaluation method have been discussed in earlier literature^19^.

**Figure 3:**
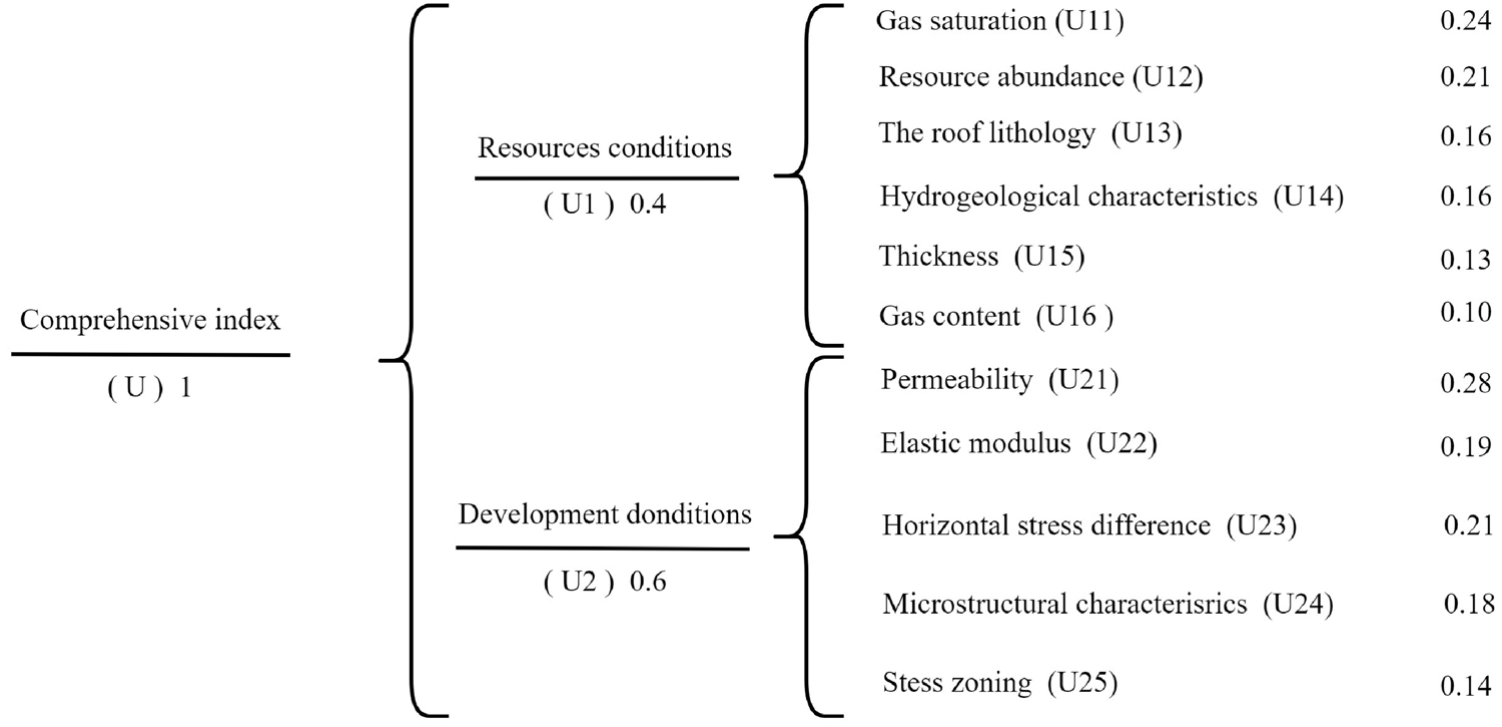
The multi-level fuzzy evaluation model with their weights.

A three-level fuzzy hierarchical quantitative evaluation system is established based on the deep CBM recoverability evaluation system established in the previous section (Fig. 3). The system aims to determine a recoverable index U (ranging from 0 to 1.0). The second level consists of two evaluation criteria: CBM resource conditions(U1) with a weight of 0.4 and CBM development conditions (U2) with a weight of 0.6. These two evaluation criteria can be further divided into 11 technically optional parameters(sub-criteria). The weights of parameters are obtained based on the measured data and experience of geologists.

## Results and discussion

### Resource characteristics of deep CBM

#### Coal thickness

**Figure 4:**
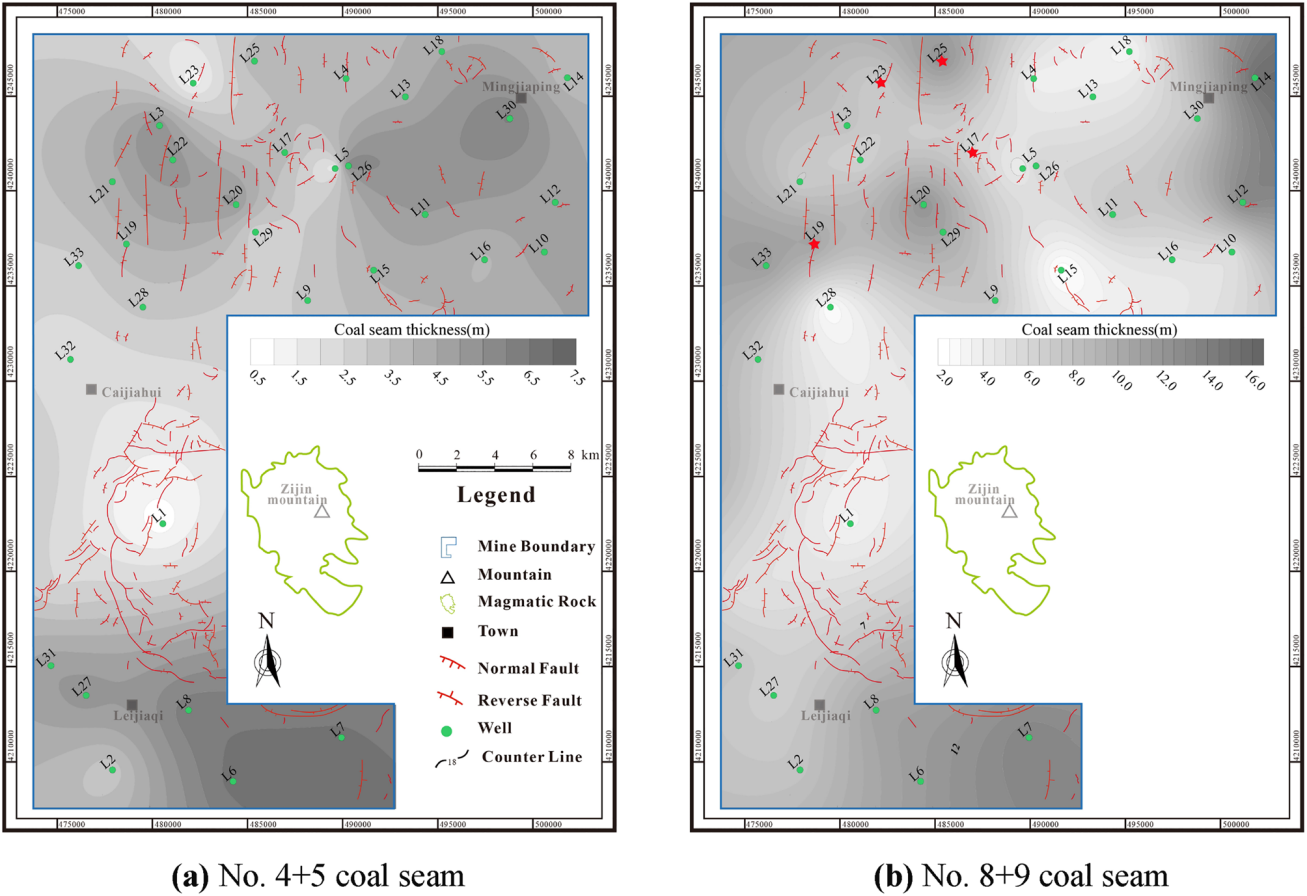
Thickness contour maps of coal seams.

The coal seams have the dual characteristics of ‘source’ and ‘reservoir’, and the thick coal seams indicate abundant CBM resources^25^. The No.4 + 5 and No.8 + 9 coal seams are widely distributed across the entire area. Specifically, the thickness of No.4 + 5 coal seam is 0.7–6.68 m (avg. 4.07 m), while thickness of No.8 + 9 coal seam is 2–15.43 m (avg. 7.72 m) (Fig. 4). Thick coal seams provide the foundation for commercial CBM development.

## Gas content and saturation

**Figure 5:**
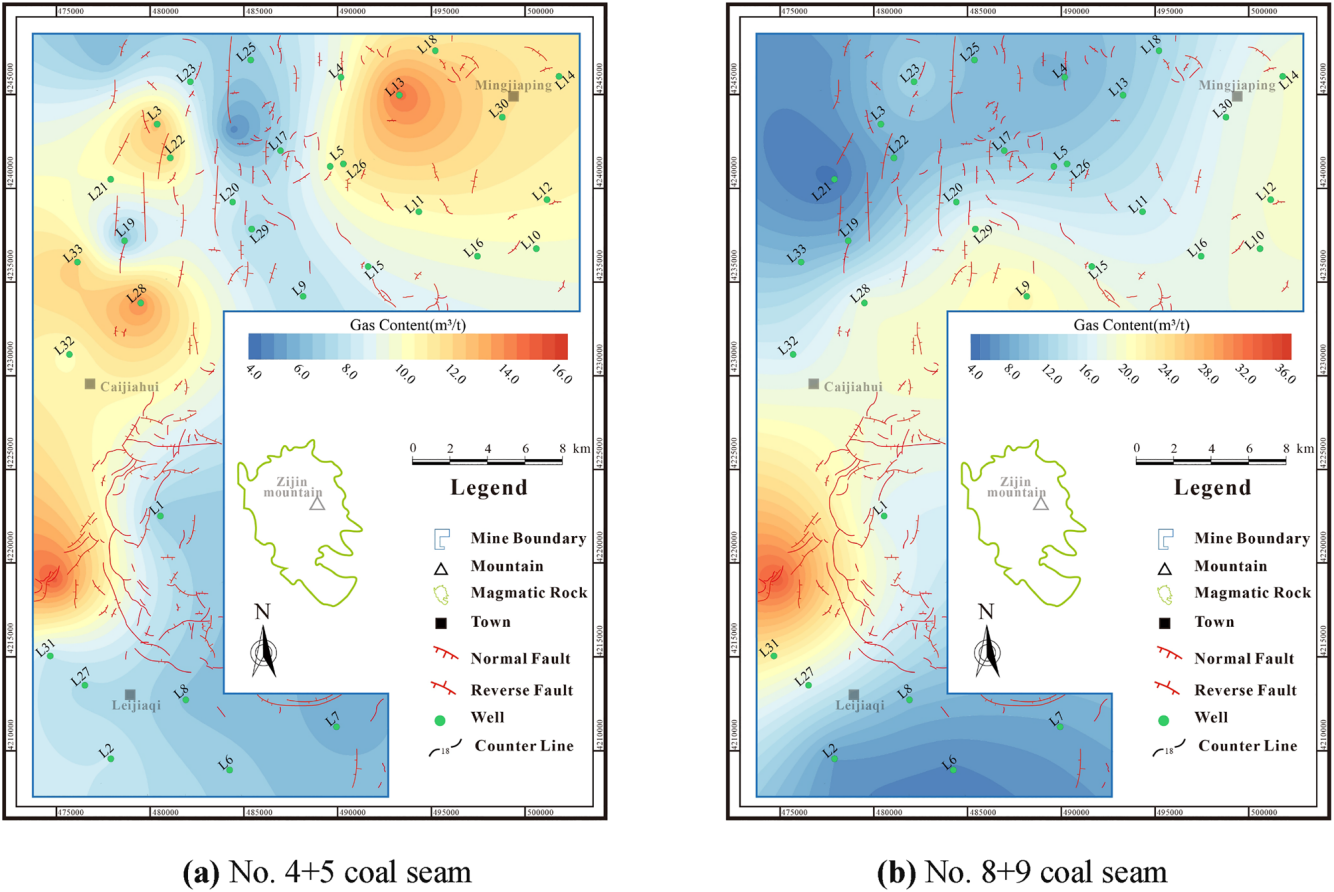
Gas content contour maps of coal seams.

The gas content of coal seam is a pivotal parameter in evaluating the potential for CBM exploration and development. This value is shaped not only by the initial gas content generation conditions but also by broader factors^13^. The gas content of No.4 + 5 coal seam is 0.41–16.39 m^3^/t (avg. 8.14 m^3^/t), while No.8 + 9 coal seam has a gas content of 3.64–32.76 m^3^/t (avg. 13.76 m^3^/t) (Fig. 5). Overall, the gas content of No.8 + 9 coal seam is significantly higher than that of No.4 + 5 coal seam, thereby signifying better resource conditions.

As CBM wells with minimal or negligible water production come into prominence, the gas saturation of a coal seam plays a pivotal role in evaluating deep CBM exploration and development^6,11^. Under in-situ conditions, reservoir pressure also determines the adsorbed gas content of coal seams except for reservoir temperature. Therefore, Eq. (1) can be used to calculate the theoretical gas content of coal seams:

1 $$ {\text{V}}_{{\text{T}}} = {\text{V}}_{{\text{L}}} {\text{P}}/\left( {{\text{P}} + {\text{P}}_{{\text{L}}} } \right) $$

The actual gas content is calculated by Eq. (2):

2 $$ {\text{V}}_{{\text{A}}} = {\text{V}}_{{\text{L}}} + {\text{V}}_{{\text{D}}} + {\text{V}}_{{\text{R}}} $$

The gas saturation is calculated by Eq. (3):

3 $$ {\text{S}}_{{\text{A}}} = {1}00\% \times {\text{V}}_{{\text{A}}} /{\text{V}}_{{\text{T}}} $$

where V_T_ is the theoretical gas content of coal seam under in-situ condition, m^3^/t, V_L_ is the Langmuir volume, m^3^/t, P is in-situ condition reservoir pressure, MPa, P_L_ is the Langmuir pressure, MPa, V_A_ is the measured gas content, m^3^/t, V_L_ is the volume of lost gas, cm^3^/g, V_D_ is the desorption gas volume, cm^3^/g, V_R_ is the residual gas volume, cm^3^/g, S_A_ is the measured gas saturation, %.

**Figure 6:**
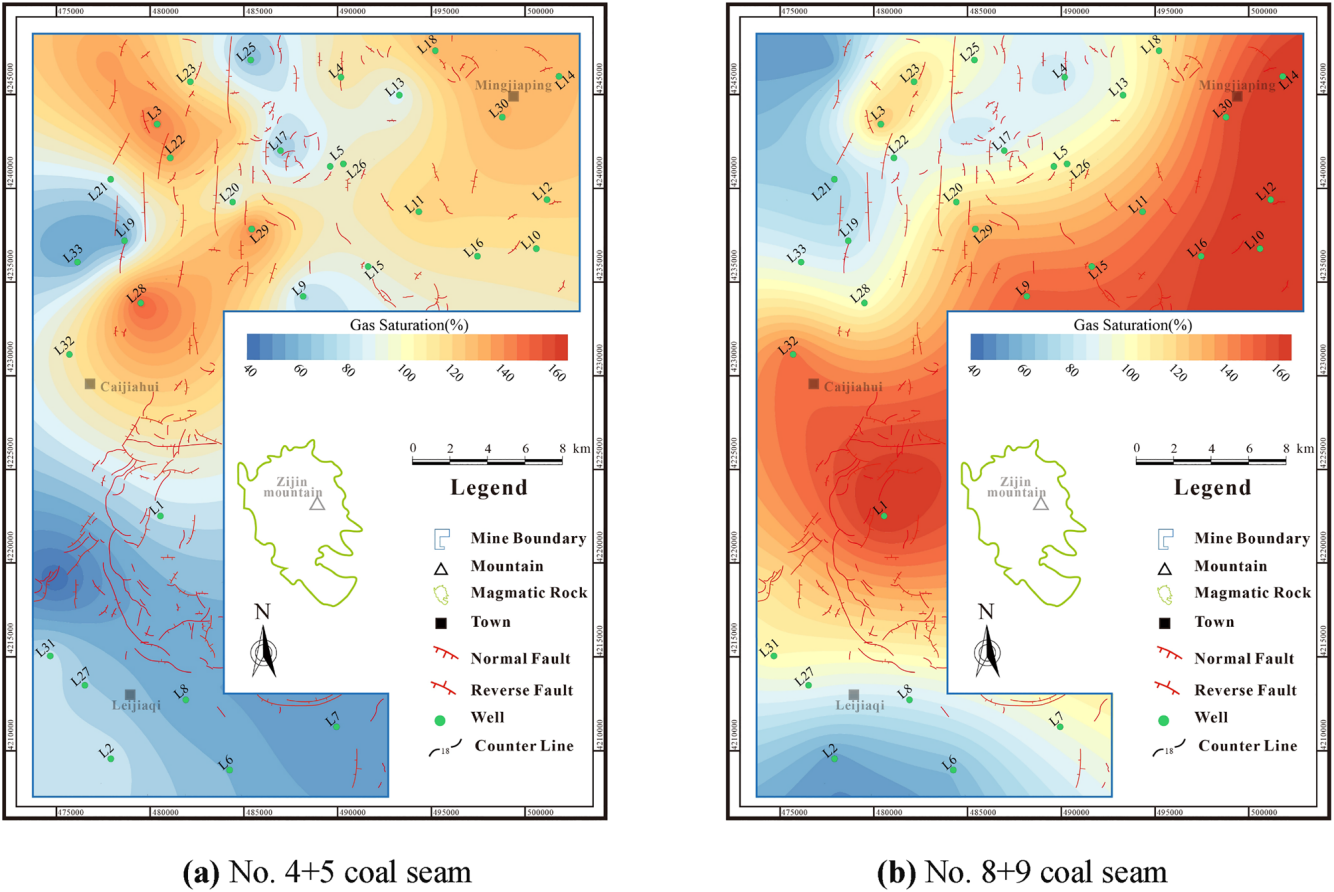
Gas saturation contour maps of coal seams.

When S_A_ is less than 100%, it indicates that the coal seam is under-saturated and has no in-situ free gas. When S_A_ is greater than 100%, the coal seam reaches saturated adsorption with in-situ free gas presented. The gas saturation of No.4 + 5 coal seam is 4.41%-148% (avg. 93.83%) (Fig. 6a), while the gas saturation of No.8 + 9 coal seam is 51.58%-167.5% (avg. 108.14%) (Fig. 6b). The No.4 + 5 oversaturated coal seam is primarily located in the northern portion of the research area, while the No.8 + 9 oversaturated coal seam is substantial developed in the research area.

## CBM resource abundance

CBM resource abundance refers to the concentration of CBM resources. This measure can be calculated in the research area by utilizing parameters such as coal seam thickness, gas content, and coal seam density.

**Figure 7:**
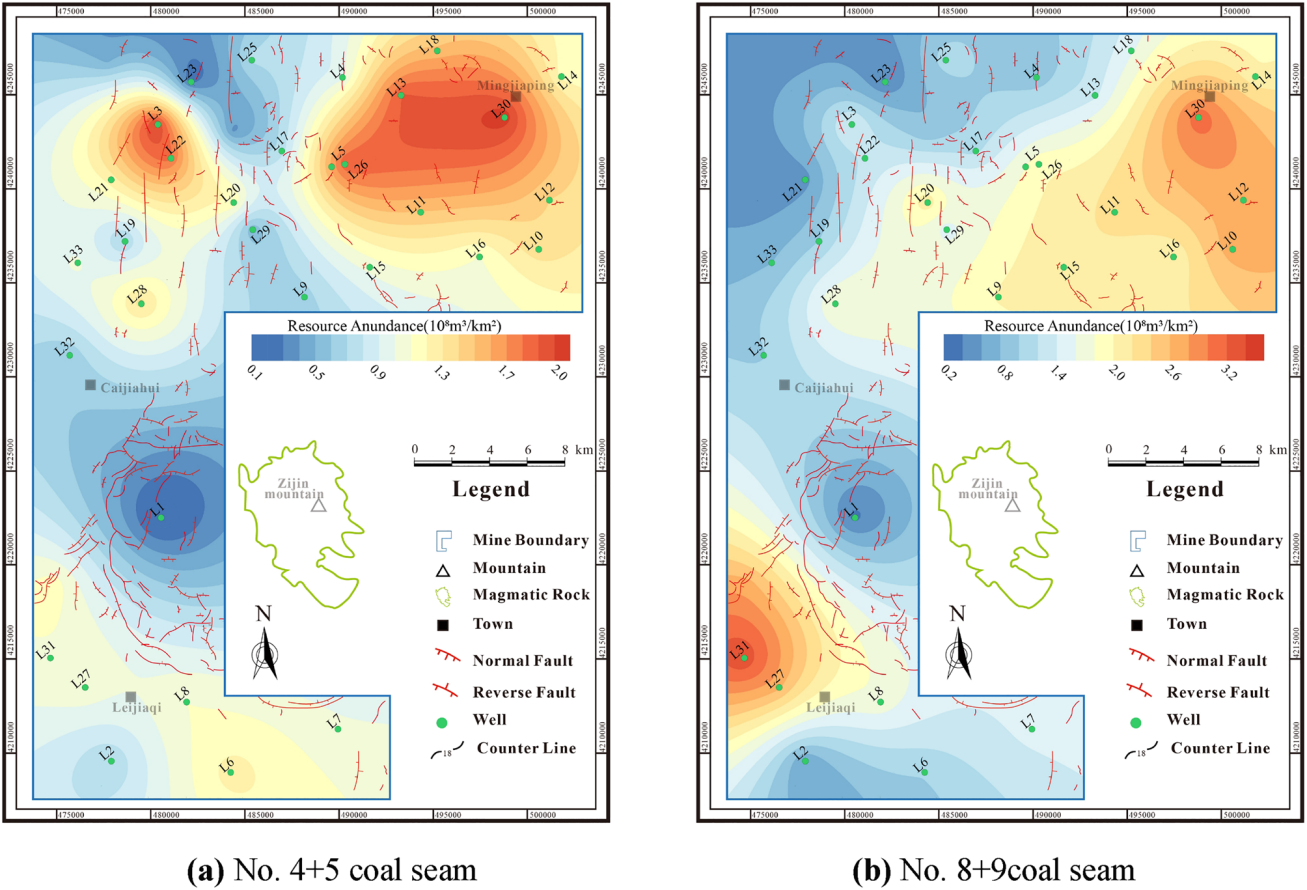
Resource abundance contour maps of coal seams.

The resources abundance of No.4 + 5 coal seam is (0.12–2.06) × 10^8^m^3^/km^2^, with an average of 1.09 × 10^8^m^3^/km^2^ (Fig. 7a). The high-value areas are primarily located in the northern and southwestern areas, with large differences in resource abundance. The resource abundance of No.8 + 9 coal seam is (0.34–3.38) × 10^8^m^3^/km^2^, with an average of 1.59 × 10^8^m^3^/km^2^(Fig. 7b), and the resource potential is better than that of No.4 + 5 coal seam.

## The roof and floor lithology and its characteristics

**Figure 8:**
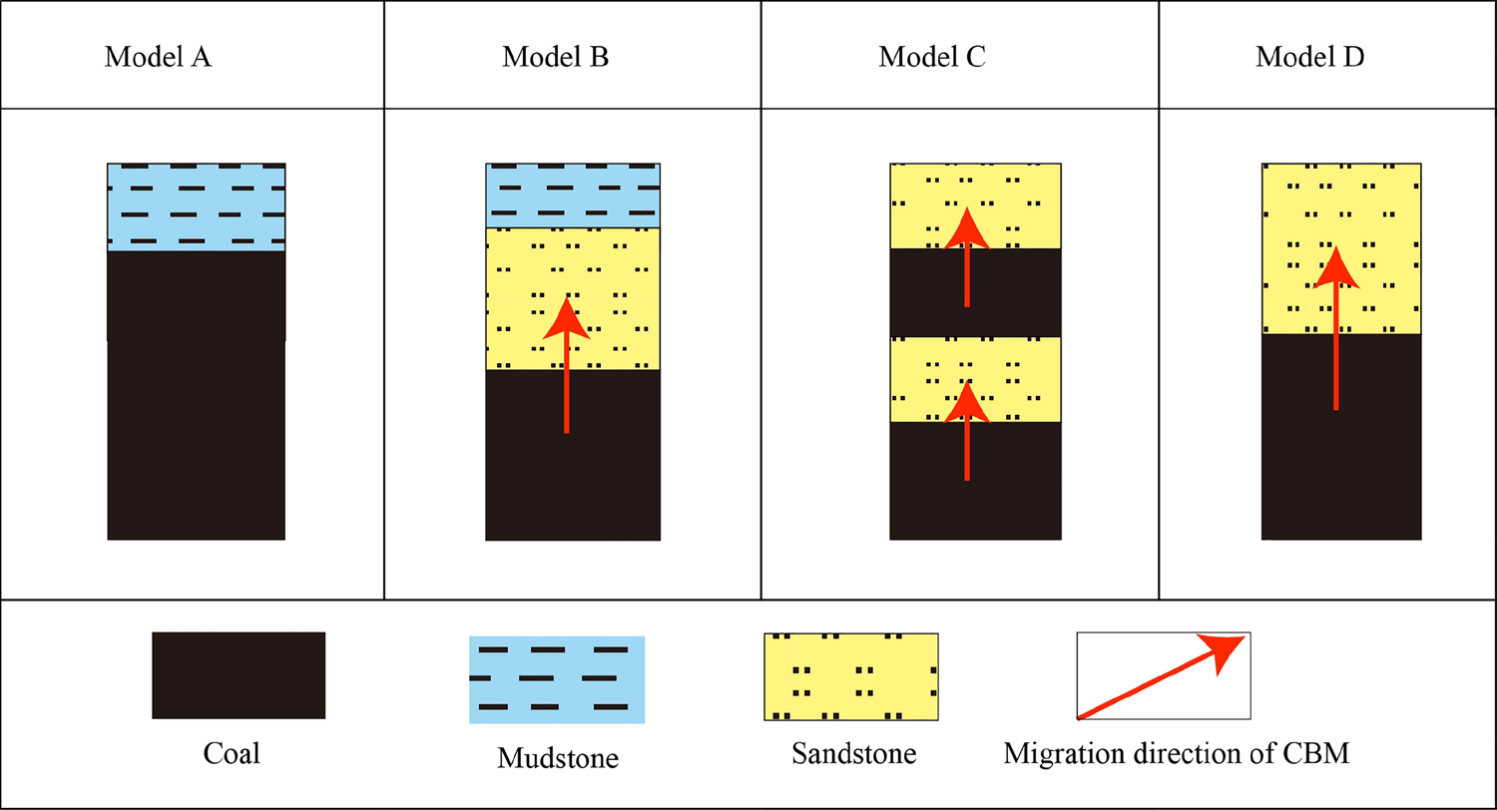
The sealing model of roof lithology of coal seams.

The sedimentary environment serves as the foundation for the enrichment and preservation of CBM. Generally, a roof with higher shale content boasts superior sealing ability, which enhances CBM preservation^13,26^. Various types of roof lithology are observed, prominently in the research area, including mudstone, sandy mudstone, fine sandstone, and medium sandstone. These lithologies give rise to several models of roof lithology sealing, leading to four distinctive types (Fig. 8). These models exert varying degrees of influence on CBM preservation. Models ‘A’ and ‘B’ are favorable to the preservation of CBM, while in Models ‘C’ and ‘D’, the tendency for CBM to escape upward is heightened, thereby hindering its enrichment and accumulation.

## Hydrogeological characteristics

**Figure 9:**
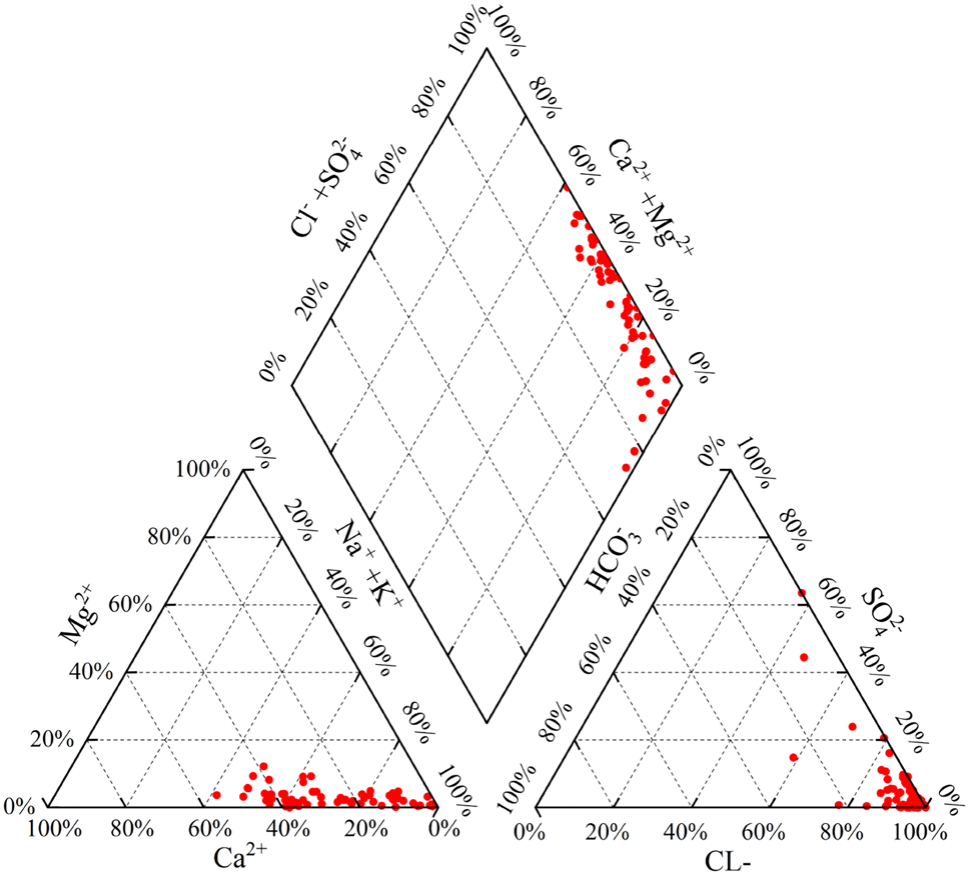
Piper Trilinear diagrams of hydrochemical compositions in coal seam water.

The salinity of coal seam water is one of the important parameters to characterize the degree of hydrodynamic activity. The high salinity indicates the stagnant water environment, which is favorable to the CBM enrichment, and serves as a fundamental geological feature for high CBM yield ^27,28^. The salinity of the formation water within the research area is 10,344-81595 mg/L (avg. 35,939.7 mg/L), which is a typical high salinity formation water. The Piper trilinear diagram based on the relative concentrations of anions and cations shows that Na^+^, and Ca^2+^ dominate the cations in the coalbed water, while the anions are dominated by Cl^-^, with higher concentrations of SO_4_^2-^ in some wells. The water types are mainly Ca–Cl and Na–Cl (Fig. 9), indicating better preservation of original depositional seawater characteristics. This reflects the stagnant water environment^29^ and provides favorable geological conditions for both CBM preservation and subsequent development.

## Development geological characteristics of deep CBM

### Permeability

Previous research has shown that coal seam permeability is an essential criterion for directly evaluating the seepage capacity of coal reservoirs, which is related to the exploitation potential and development efficiency of CBM^13^. The well test permeability in the research area varies from 0.008 to 2.29 mD (avg. 0.33mD), which is a typical low permeability reservoir (Table 1).

**Table 1:** The well test permeability of Linxing Block.

Well	Test method	Depth	Coal	Permeability/mD
LX101	Injection/fall-off well test	1216	8 + 9	0.32
LX102	Injection/fall-off well test	1086.85	8 + 9	0.3
LX103	Injection/fall-off well test	649.52	4 + 5	0.42
LX104	Injection/fall-off well test	950.72	8 + 9	0.62
LX105-1	Injection/fall-off well test	816.15	4 + 5	0.41
LX105-2	Injection/fall-off well test	876.28	8 + 9	0.185
LX106	Injection/fall-off well test	1684.44	8 + 9	0.008
LX107	Injection/fall-off well test	2064	–	0.06
LX108	Injection/fall-off well test	2016	–	0.65

Because the large-scale CBM exploitation has not been realized in the Linxing Block, the availability of measured data on coal seam physical properties is limited. Thus, the utilization of effective methods to calculate coal seam permeability becomes imperative. At present, there are numerous approaches to obtain coal seam permeability^30,31^. Among them, the application of geophysical logging stands out due to its economic feasibility and convenience^32,33^. Following the principles of logging interpretation, the permeability of coal seam was calculated. The permeability of deep coal seam in Linxing Block is generally low (0.001–0.62 mD, avg.0.136 mD). The extremely low permeability makes the deep CBM development dependent on the effective transformation of coal reservoirs.

## Mechanical parameters characteristics of deep coal seam

In addition to the external stress conditions, the mechanical parameters of coal seam also have a substantial influence on the hydraulic fracturing results. The main parameters including elasticity, strength, deformation, and fracture characteristics, directly shape the hydraulic fractures in terms of extension and expansion. According to Chen et al.,^34^ under the same stress conditions, higher elastic modulus values in coal seams result in reduced deformations, consequently hindering the propagation of hydraulic fractures. In addition, greater tensile strength indicates the ability of coal seam to withstand more stress before failure, a characteristic that impedes the initiation and propagation of hydraulic fractures^35^. The Young’s modulus of No.4 + 5 coal seam is 13.25–29.58 GPa (avg. 18.55 GPa), and that of No.8 + 9 coal seam is 12.58–34.52 GPa (avg. 20.07 GPa). And with the increasement of Young’s modulus of coal seam, the hydraulic fractures tend to form high, narrow, and short fractures, and the fractures are easy to extend in the vertical direction. The scale of hydraulic fracturing is small, which is unfavorable to the development of deep CBM^35^.

## In-situ stress distribution characteristics

The geological characteristics of deep coal reservoirs are more complicated, and the influence of in-situ stress on the physical properties and deep CBM development is becoming more and more obvious. The impact of in-situ stress on the physical properties of coal reservoirs is primarily manifested in the opening/closing of coal cleats/fractures, thus limiting the exploitation of CBM^36^. In addition, at large depths, the complexity of in-situ stress within coal reservoirs intensifies, leading to substantial alterations in stress states. Consequently, the difficulty of hydraulic fracturing in deep coal reservoirs increases exponentially. This phenomenon is evidenced by factors such as stress zoning and horizontal stress discrepancies, both of which play a substantial role in hydraulic fracture propagation^37^, which has been verified in Daning-jixian Block and Yanchuannan Block^6,38^. Therefore, the in-situ stress characteristics of coal reservoir are of significant to the evaluation of CBM.

Considering status of the Linxing Block as a nascent frontier for deep CBM exploration and development, the paucity of hydraulic fracturing data poses a challenge in characterizing the spatial variation of the in-situ stress field. Therefore, an in-situ stress calculation model that takes into account rock mechanical properties and tectonic strains is employed to estimate the horizontal maximum stress (*S*_*Hmax*_) and horizontal minimum stress (*S*_*hmin*_)^21,39^.

4 $$ S_{H\max } = \frac{{\mu_{S} }}{{1 - \mu_{S} }}(S_{V} - \alpha P_{0} ) + \alpha P_{0} + \frac{{E_{S} }}{{1 - \mu_{S}^{2} }}\xi_{H} + \frac{{E_{S} \mu_{S} }}{{1 - \mu_{S}^{2} }}\xi_{h} $$

5 $$ S_{h\min } = \frac{{\mu_{S} }}{{1 - \mu_{S} }}(S_{V} - \alpha P_{0} ) + \alpha P_{0} + \frac{{E_{S} }}{{1 - \mu_{S}^{2} }}\xi_{h} + \frac{{E_{S} \mu_{S} }}{{1 - \mu_{S}^{2} }}\xi_{H} $$

where *μ*_*S*_ is the static Poisson ratio, *S*_*V*_ is the vertical principal stress, *α* is the Biot coefficient, *E*_*S*_ is the static Young’s modulus, GPa, *ξ*_*h*_ is the strain in the horizontal minimum stress direction, *ξ*_*H*_ is the strain in the horizontal maximum stress direction.

The *S*_*V*_ can be obtained by integrating the density log^40^.

6 $$ S_{V} = \int_{0}^{H} {\rho (H)} gdH $$

where *ρ* is the formation density, *H* is the depth.

**Figure 10:**
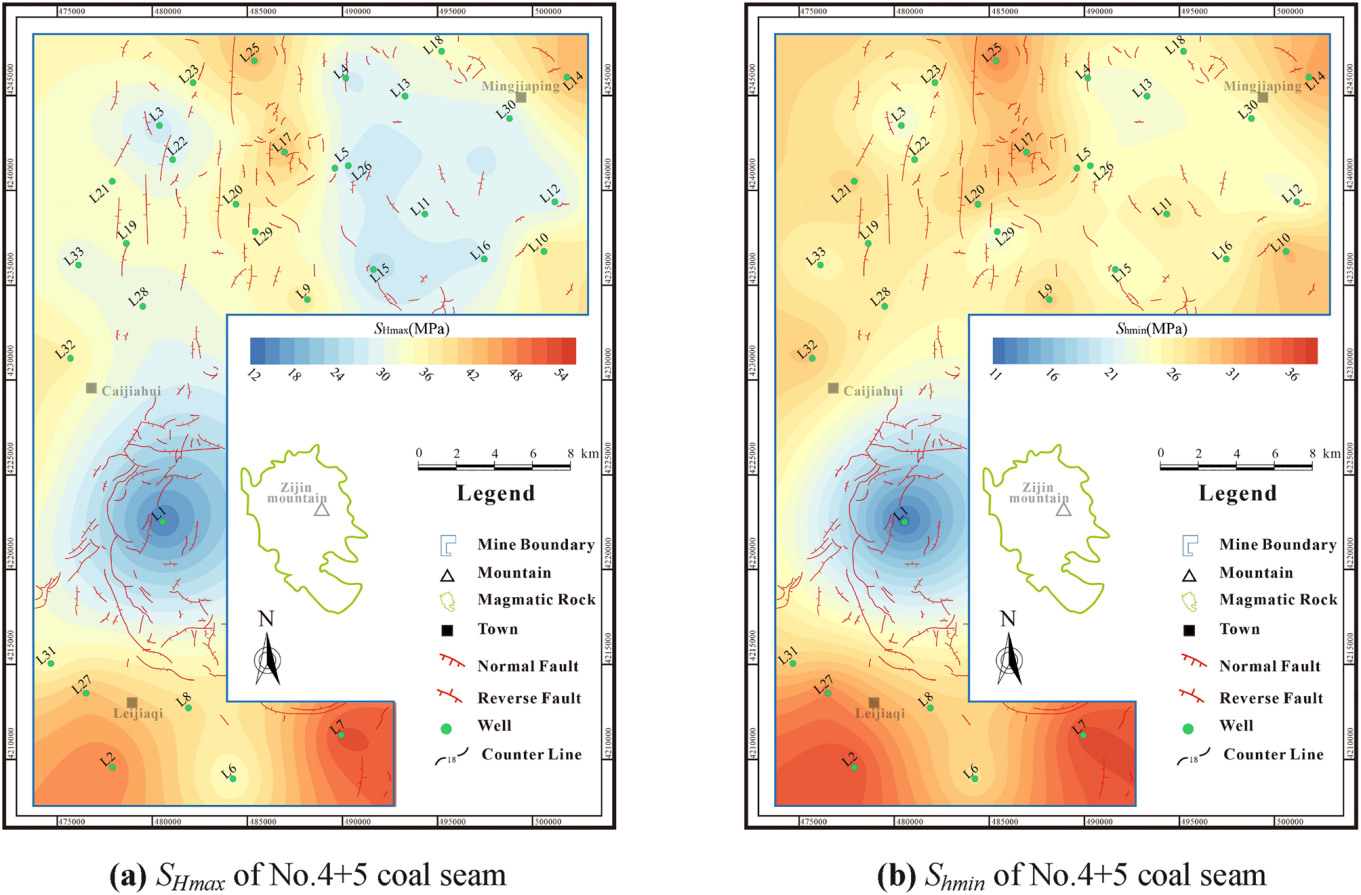
The In-situ stress distribution characteristics of No. 4 + 5 coal seam.

**Figure 11:**
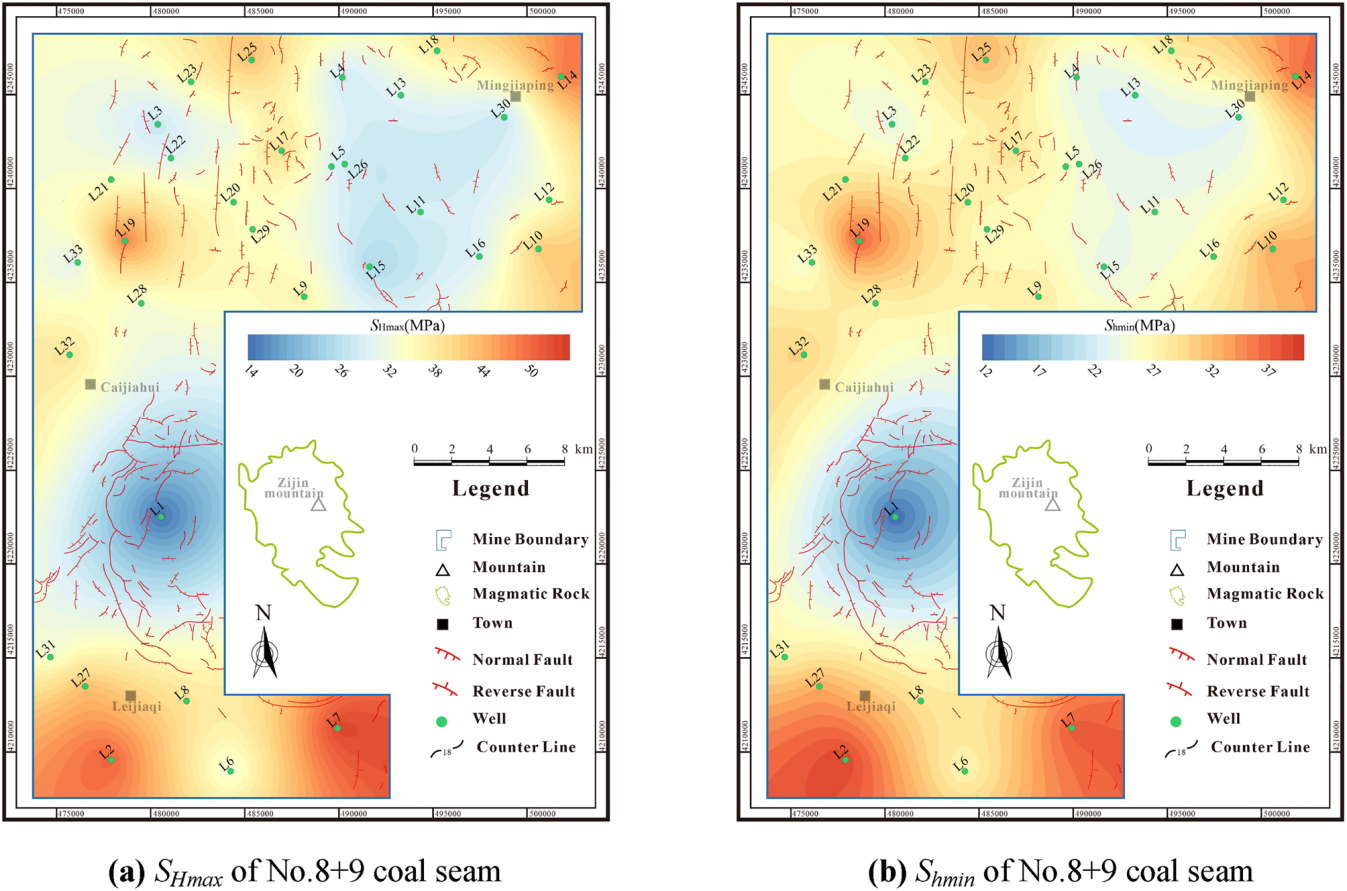
The In-situ stress distribution characteristics of No. 8 + 9 coal seam.

The *S*_*hmin*_ of No.4 + 5 coal seam is 11.33–35.49 MPa (avg.26.09 MPa), the *S*_*Hmax*_ of No.4 + 5 coal seam is 13.57–51.84 MPa (avg.33.01 MPa) (Fig. 10). The *S*_*hmin*_ of No.8 + 9 coal seam is 11.98–38.99 MPa (avg.27.79 MPa), the *S*_*Hmax*_ No.8 + 9 coal seam is 14.23–53.55 MPa (avg.34.77 MPa) (Fig. 11). According to the judging criteria^41^, the research area is in the strong stress region (*S*_*hmin*_ is 10–18 MPa) and super strong region (*S*_*hmin*_ is greater than 30 MPa), which is easy to cause the fracture closure and stress sensitivity damage to the reservoir.

Additionally, the HSD is a key parameter to determine the performance of the fracture transformation. A higher HSD is correlated with an increased likelihood of forming uncomplicated fractures^42,43^.Gao et al., claimed that when the HSD in Linxing Block is less than 3 MPa, the hydraulic fractures propagate along the direction of the natural fractures^44^. That is, when the HSD is minimal, hydraulic fractures tend to propagate along the weak surface. When the HSD is greater than 3 MPa, hydraulic fractures gradually transform from the natural fracture direction to the *S*_*Hmax*_ direction. When the HSD exceeds 6 MPa, the extension direction of hydraulic fractures completes the transition and propagates along the direction of *S*_*Hmax*_. Moreover, as the HSD continues to increase, the hydraulic fractures become concentrated in the direction of *S*_*Hmax*_, ultimately culminating in the formation of a simple fracture extending exclusively in the direction of *S*_*Hmax*_ when the HSD is greater than 10 MPa. Evidently, an HSD of less than 6 MPa is conducive to form a complex fracture network, while more than 10 MPa tends to form simple fractures. However, the HSD in Linxing Block ranges from 3.78 to 16.78 MPa (avg. 7.67 MPa). The high stress anisotropy contributes to forming simple hydraulic fractures. The complex and high-stress in-situ conditions within the Linxing Block necessitate elevated standards for deep coal reservoir transformation techniques.

## Structural characteristics

Structural characteristics are widely recognized as the most influential geological factor in the enrichment of CBM, determining both its generation and preservation^45^. Yan et al., believed that the deep microstructural characteristics also play a key role in the exploitation of CBM, especially in the positive microstructure^6^. Due to the development of tensile fractures and high permeability in positive microstructures, the coal seam is easily fractured, and it tends to form well-connected fracture network systems after hydraulic fracturing, which promotes the efficient exploitation of CBM.

**Figure 12:**
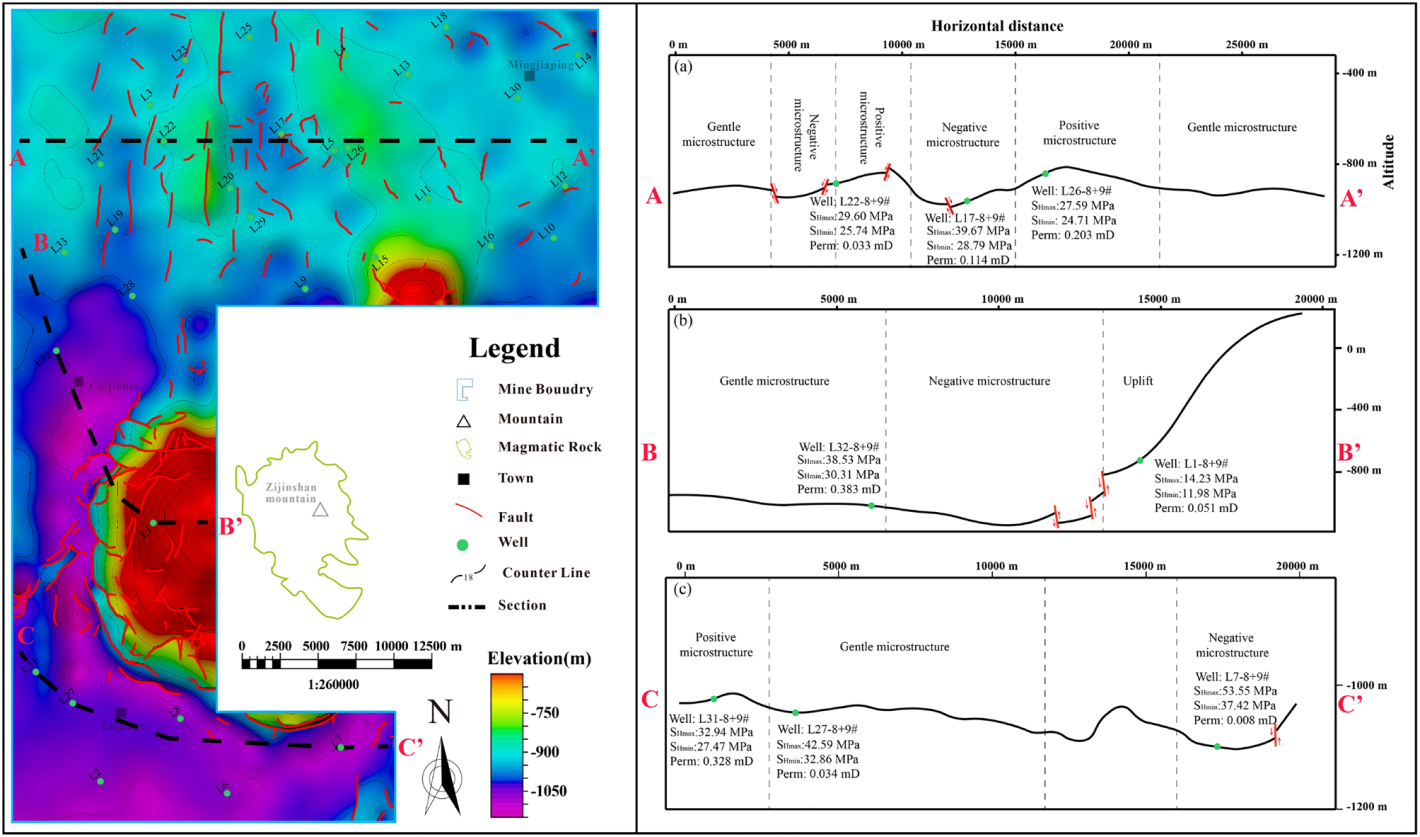
Structural characteristics of coal seam.

Within the research area, various microstructures including positive microstructure, gentle microstructure, negative microstructure, and uplift (Fig. 12). In the northern region, the positive microstructure and gentle microstructure are more developed, and the in-situ stress is low (Fig. 12a), which is favorable for CBM development. Conversely, in the central area, magmatic intrusion gives rise to uplift and a negative microstructure centered around the uplift (Fig. 12b, c), resulting in higher in-situ stress. The southern area is characterized by simpler structural patterns and the prevalence of gentle microstructures (Fig. 12c). Despite this, it still contends with high in-situ stress levels due to significant burial depths, making effective coal reservoir transformation challenging.

## Evaluation results and favorable area classification

### Multi-level fuzzy evaluation results

The recoverable potential of deep CBM is affected by complex geological factors. A simple evaluation model cannot accurately reflect this potential. Therefore, a three-level fuzzy hierarchical quantitative evaluation method is established using the multi-level fuzzy evaluation method. The model is used to systematically evaluate the recoverability of deep CBM in the research area. The multi-level fuzzy quantitative evaluation process includes the following steps:

First, optimization of key parameters. In this study, 11 geological parameters were selected as the evaluation indicators in the multi-level fuzzy evaluation model (Fig. 3), mainly including gas saturation(U11), Resource abundance(U12), the roof lithology(U13), hydrogeologic characteristics(U14), thickness(U15), gas content(U16), permeability(U21), stress zoning(U22), horizontal stress difference(U23), microstructural characteristics(U24), elastic modulus(U25).

Second, determining Parameters Weights. To determine the importance of different parameters, a discriminant matrix is established. The eigenvector and maximal characteristic root(*λ*_*max*_) of the discriminant matrix are then computed using MATLAB software (version 9.6), designed by MathWorks corporation^46^. The eigenvector of the discriminant matrix can determine the weight of each key parameter (Table 2). Additionally, it is necessary to conduct a consistency test to verify the correctness and credibility of the calculated resultss^13,17^.

**Table 2:** The discriminant matrix of the key parameters.

	Discriminant matrix of key parameters							Wight	*λ*_*max*_	(*C.R.*/%)
U-U1	U1	U11	U12	U13	U14	U15	U16	W-U1	6.08	3.5 < 10
	U11	1	1.5	1.67	1.43	2	1.9	0.24		
	U12	0.67	1	1.67	1.43	1.7	1.79	0.21		
	U13	0.6	0.6	1	1.2	1.55	1.67	0.16		
	U14	0.7	0.7	0.83	1	1.46	1.58	0.16		
	U15	0.5	0.59	0.65	0.68	1	1.78	0.13		
	U16	0.53	0.56	0.6	0.63	0.56	1	0.10		
U-U2	U2	U21	U22	U23	U24	U25		W-U2	5.049	1.09 < 10
	U21	1	1.25	1.79	1.2	2		0.28		
	U22	0.8	1	0.99	1.1	1.11		0.19		
	U23	0.56	1.01	1	1.4	1.61		0.21		
	U24	0.83	0.91	0.71	1	1.2		0.18		
	U25	0.5	0.9	0.62	0.83	1		0.14		

Third, calculation parameters membership. By calculating the membership of different parameters, the parameter normalization can be realized and the parameters can be analyzed using a unified standard. However, there are slight differences in determining the membership of qualitative and quantitative indicators.

Quantitative indicators include gas saturation(U11), resource abundance(U12), coal seam thickness(U15), gas content(U16), permeability(U21), elastic modulus(U22), stress zoning(U23), and horizontal stress difference(U24). These indicators’ memberships are typically determined using linear piecewise functions. For instance, the HSD plays a critical role in controlling fracture extension during hydraulic fracturing. When the HSD is less than 6 MPa, it tends to form complex fracture networks. When the HSD is over 10 MPa, it tends to form single fractures, impeding the extensive desorption, diffusion, and seepage of CBM^44^. Therefore, the membership function of the HSD is set (Eq. 14). Similar methods are used to construct the membership function/equation of other indicators.

7 $$ U11 = \left\{ {\begin{array}{*{20}c} 0 & {S_{g} \le 60} \\ {0.045S_{g} - 2.6} & {{60 < }S_{g} \le 80} \\ 1 & {S_{g} > 80} \\ \end{array} } \right. $$

8 $$ U12 = \left\{ {\begin{array}{*{20}c} 0 & {A \le 1} \\ {2A - 2} & {1 < A \le 1.5} \\ 1 & {A > 1.5} \\ \end{array} } \right. $$

9 $$ U15 = \left\{ {\begin{array}{*{20}c} {0.2} & {T \le 2} \\ {0.4M - 0.6} & {2 < T \le 4} \\ 1 & {T > 4} \\ \end{array} } \right. $$

10 $$ U16 = \left\{ {\begin{array}{*{20}c} {0.2} & {G \le 4} \\ {0.2G - 0.6} & {4 < G \le 8} \\ 1 & {G > 8} \\ \end{array} } \right. $$

11 $$ U21 = \left\{ {\begin{array}{*{20}c} {0.2} & {P_{erm} \le 0.1} \\ {5P - 0.5} & {0.1 < P_{erm} \le 0.3} \\ 1 & {P_{erm} > 0.3} \\ \end{array} } \right. $$

12 $$ U22 = \left\{ {\begin{array}{*{20}c} {0.4} & {E \le 15} \\ {0.12E - 1.4} & {15 < E \le 20} \\ 1 & {E > 20} \\ \end{array} } \right. $$

13 $$ U23 = \left\{ {\begin{array}{*{20}c} 1 & {S \le 18} \\ { - 0.05B + 1.9} & {18 < S \le 3} \\ {0.2} & {S > 30} \\ \end{array} } \right.0 $$

14 $$ U24 = \left\{ {\begin{array}{*{20}c} 1 & {H \le 6} \\ { - 0.2H + 2.2} & {6 < H \le 10} \\ {0.2} & {H > 10} \\ \end{array} } \right. $$

Qualitative indicators include the roof lithology (U13), hydrogeological characteristics(U14), and microstructural characteristics (U25), the membership of which are quantified based on geological data and research results (Table 3).

**Table 3:** The membership of qualitative indicators.

Microstructural characteristics(U25)	The roof lithology(U13)	Hydrogeologic characteristics(U14)
Positive microstructure (0.6–1)	Mudstone (0.8–1)	Retention zone (0.6–1)
Gentle microstructure (0.4–0.6)	Sandy mudstone (0.6–0.8)	Transition zone (0.2–0.6)
Negative microstructure (0–0.4)	Fine sandstone (0.2–0.6)	
Uplift (0)	Medium sandstone (0–0.2)	Runoff zone (0–0.2)

Finally, a three-level fuzzy quantitative evaluation model is established (Table 4). Through the evaluation model, the recoverability evaluation score U (ranging from 0 to 1.0) can be calculated. The higher value of U, the better the prospects for CBM exploration and development.

**Table 4:** The quantitative evaluation modeling of deep CBM recoverable favorable areas.

Recoverable index	Two-level indicators	Three-level indicators	Weight	Membership	Weight coefficient
Ui(U)	Ui(U1)	Ui(U11)	0.24	Ei1	0.24 × Ei1
		Ui(U12)	0.21	Ei2	0.21 × Ei2
		Ui(U13)	0.16	Ei3	0.16 × Ei3
		Ui(U14)	0.16	Ei4	0.16 × Ei4
		Ui(U15)	0.13	Ei5	0.13 × Ei5
		Ui(U16)	0.10	Ei6	0.10 × Ei6
	Ui(U2)	Ui(U21)	0.28	Fi1	0.28 × Fi1
		Ui(U22)	0.19	Fi2	0.19 × Fi2
		Ui(U23)	0.21	Fi3	0.21 × Fi3
		Ui(U24)	0.18	Fi4	0.18 × Fi4
		Ui(U25)	0.14	Fi5	0.14 × Ni5

## Favorable areas optimization of deep CBM

### Resource conditions of deep CBM

**Figure 13:**
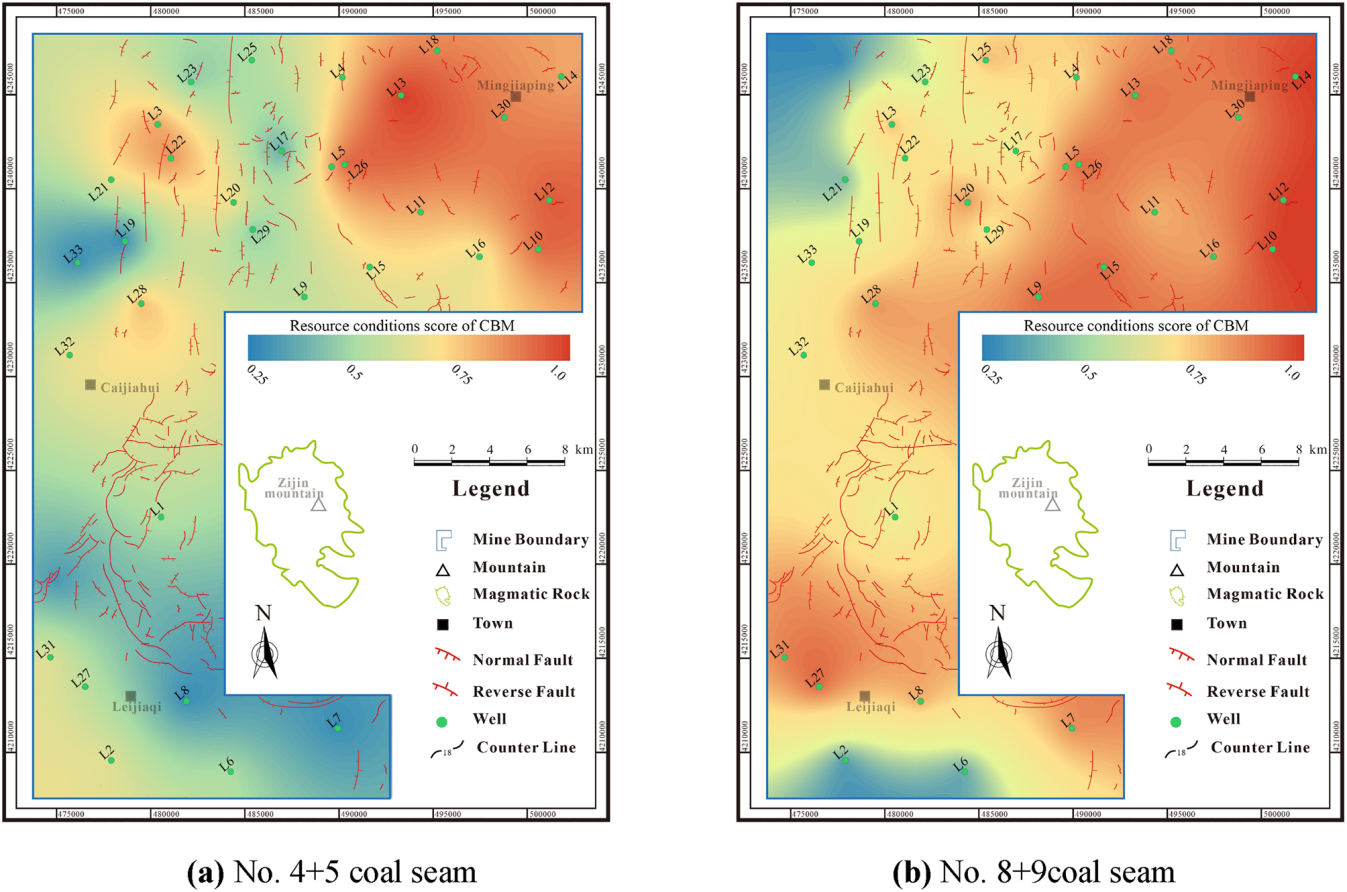
Resource conditions score contour maps of coal seams.

Resource conditions are the key to CBM exploitation. The comprehensive score of No.4 + 5 coal seam resource conditions is 0.28–0.98 (avg. 0.63) (Fig. 13a). While the comprehensive score of No.8 + 9 coal seam resource conditions is 0.27–0.92 (avg. 0.74) (Fig. 13b), which is superior to that of No.4 + 5 coal seam. The favorable areas of No.4 + 5 coal seam resources are primarily located in the northeastern area, while the favorable areas of No.8 + 9 coal seam resources are mainly in the northeastern and southern areas. Overall, the research area boasts promising resource potential, and the No.8 + 9 coal seam is more favorable than the No.4 + 5 coal seam.

## Development conditions of deep CBM

**Figure 14:**
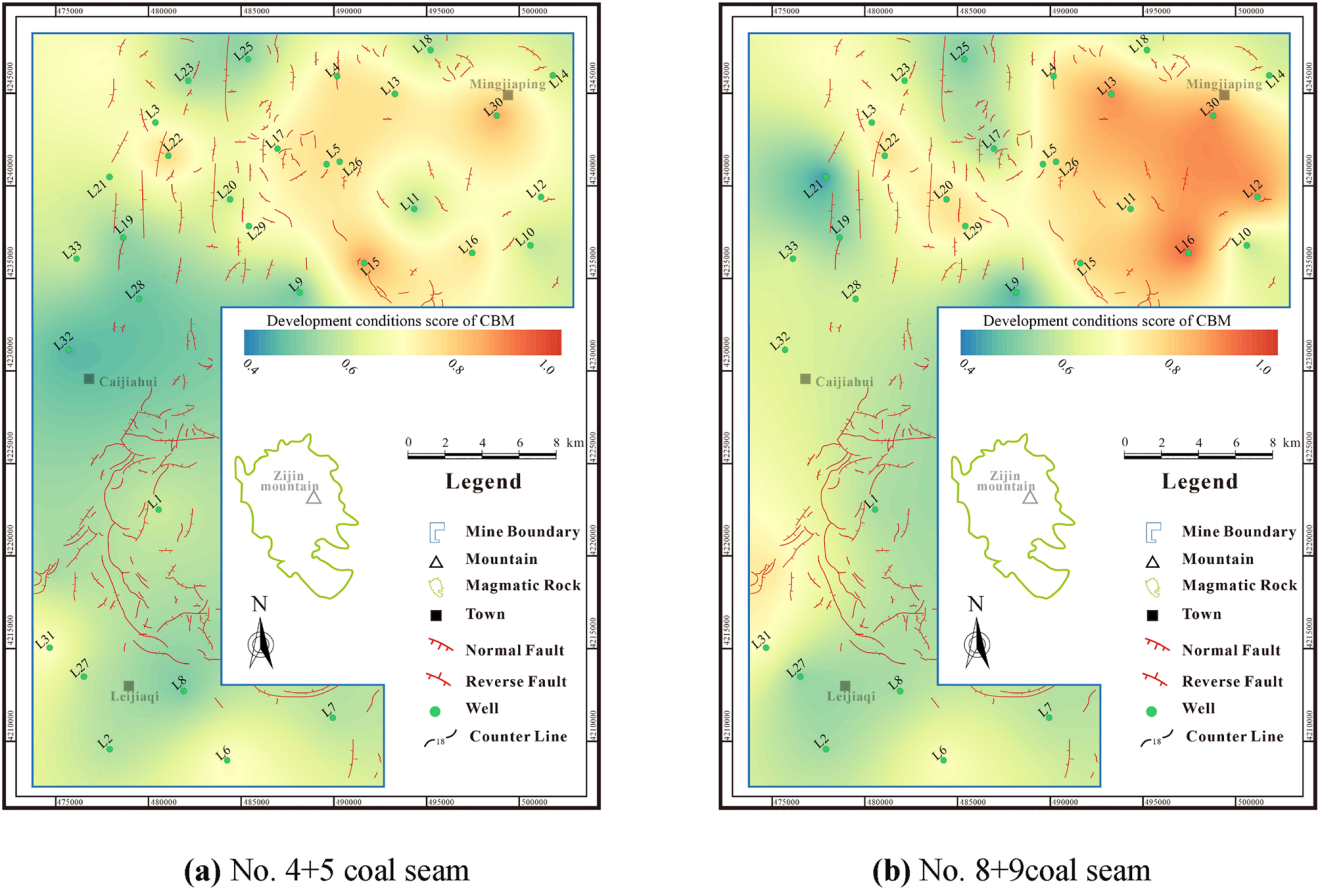
Development conditions score contour maps of coal seams.

Compared with the resource conditions, the development conditions are the key to the commercial exploitation of CBM. The comprehensive score of No.4 + 5 coal seam development conditions is 0.27–0.83 (avg. 0.52), while the comprehensive score of No.8 + 9 coal seam development conditions is 0.28–0.88 (avg. 0.56). The favorable areas for development of the two sets of coal seams are primarily distributed in the northeastern area, while the development conditions in the southern area are relatively poor (Fig. 14). This underscores the necessity for elevated proficiency in deep coal reservoir transformation techniques to offset these challenges.

## Recoverable favorable area of deep CBM

**Figure 15:**
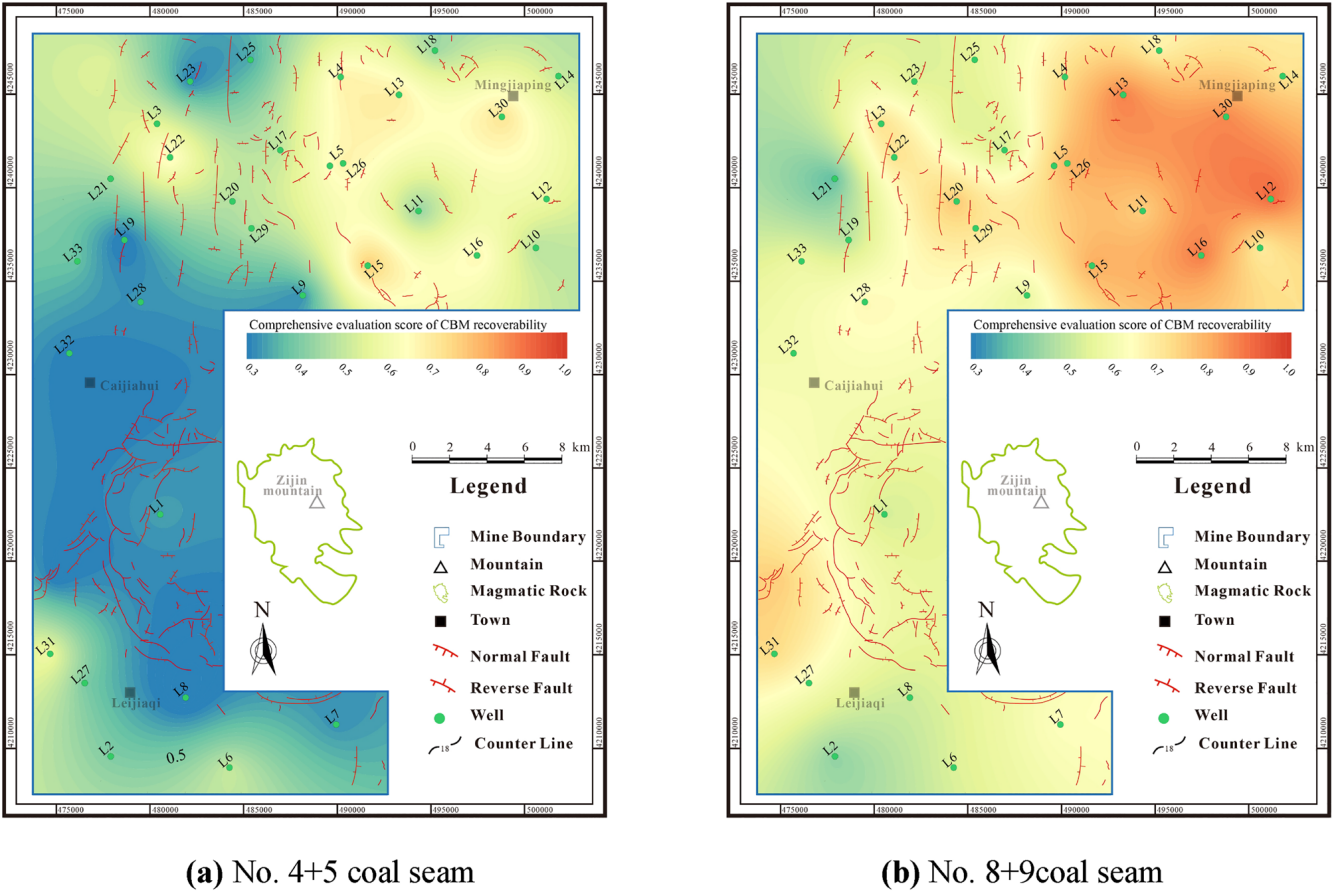
Comprehensive evaluation score contour maps of coal seams.

Based on the classification standard of recoverable favorable areas in Fig. 2, the comprehensive score of the recoverable favorable area for No.4 + 5 coal seam varies from 0.36 to 0.81 (avg. 0.56). It is dominated by Level IV area, which is primarily dispersed in the central-southern and northern portions of the research area, followed by Level III area, which is primarily located in the northern portion of the research area. The main CBM development area, known as the Level II area, is situated in the northeast of the research area (Fig. 15a). The comprehensive score of No.8 + 9 coal seam recoverable favorable area is 0.31 to 0.91 (avg. 0.63). It is also dominated by Level IV area, which is primarily located in the northwestern, central, and southern parts of the research area. Level I and II areas are followed, located in the northeastern part of the research area (Fig. 15b). In general, the No.8 + 9 coal seam shows promising development prospect.

**Figure 16:**
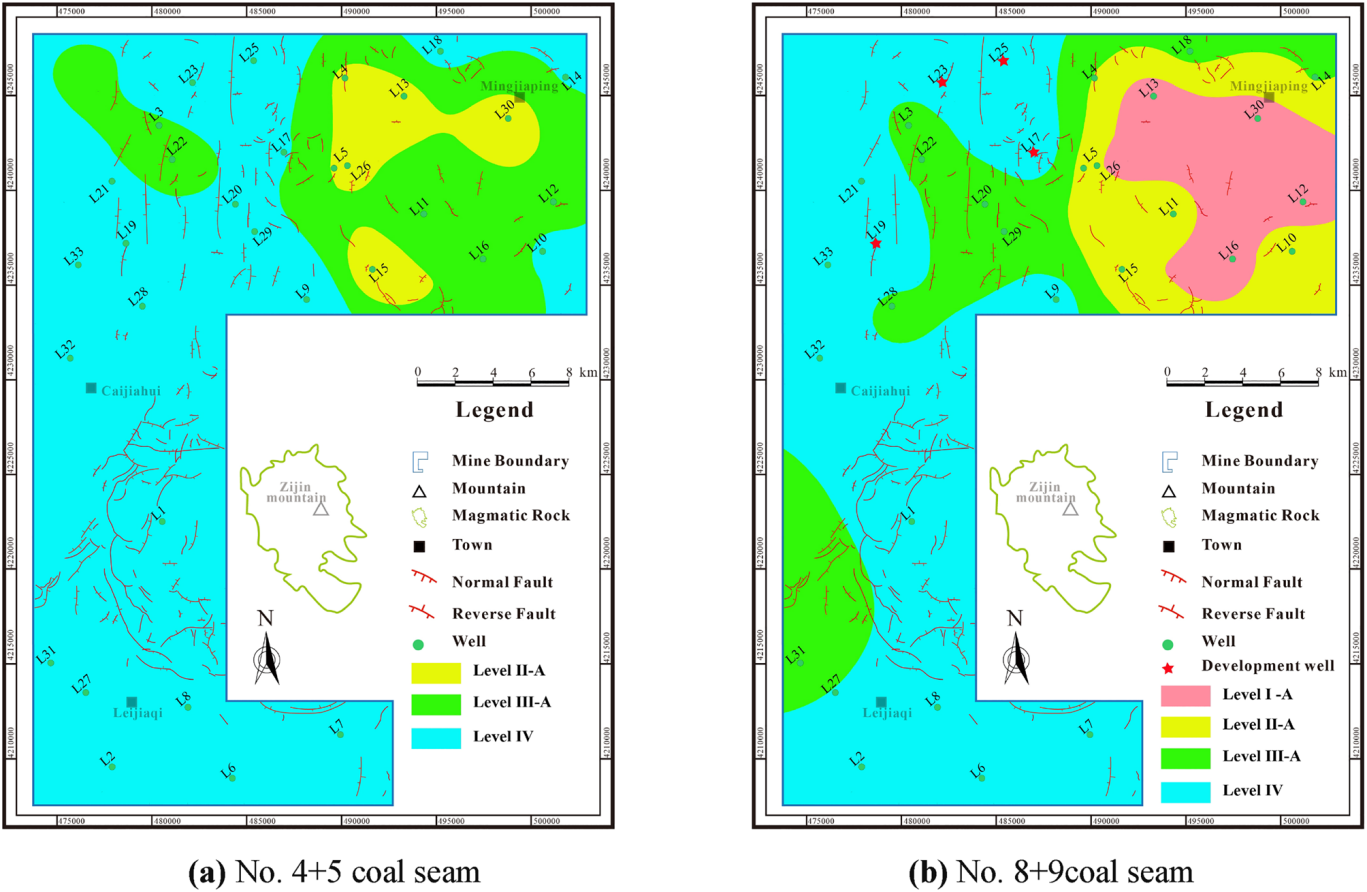
Recoverable favorable area contour maps of coal seams.

A comprehensive analysis indicates that Level I areas in the research area generally have better resource and development conditions, with development condition scores exceeding 0.8. These areas have less difficulty in hydraulic fracturing of coal reservoirs, and it is easy to form high permeability and high yield potential. The resource condition score of Level II areas is greater than 0.7, which is the main development area in the research area. Additionally, the resource condition score of Level III areas is greater than 0.6. However, the efficient development of deep CBM is hindered by complex geological conditions. For instance, high in-situ stress and low physical properties make hydraulic fracture difficult and do not promote high production. The Level III areas are used as the undertaking area of production of deep CBM and have better exploration prospects. The Level IV areas are widely distributed in the research area. Due to its poor resource conditions (score less than 0.6) and difficult hydraulic fracturing, it is not conducive to CBM development. The resource conditions in the favorable area are generally superior to the development conditions, and these favorable areas are classified as Class A (Fig. 16). The reservoir transformation is relatively difficult and prone to the characteristics of CBM enrichment but not high production.

## Reliability verification of evaluation system

**Figure 17:**
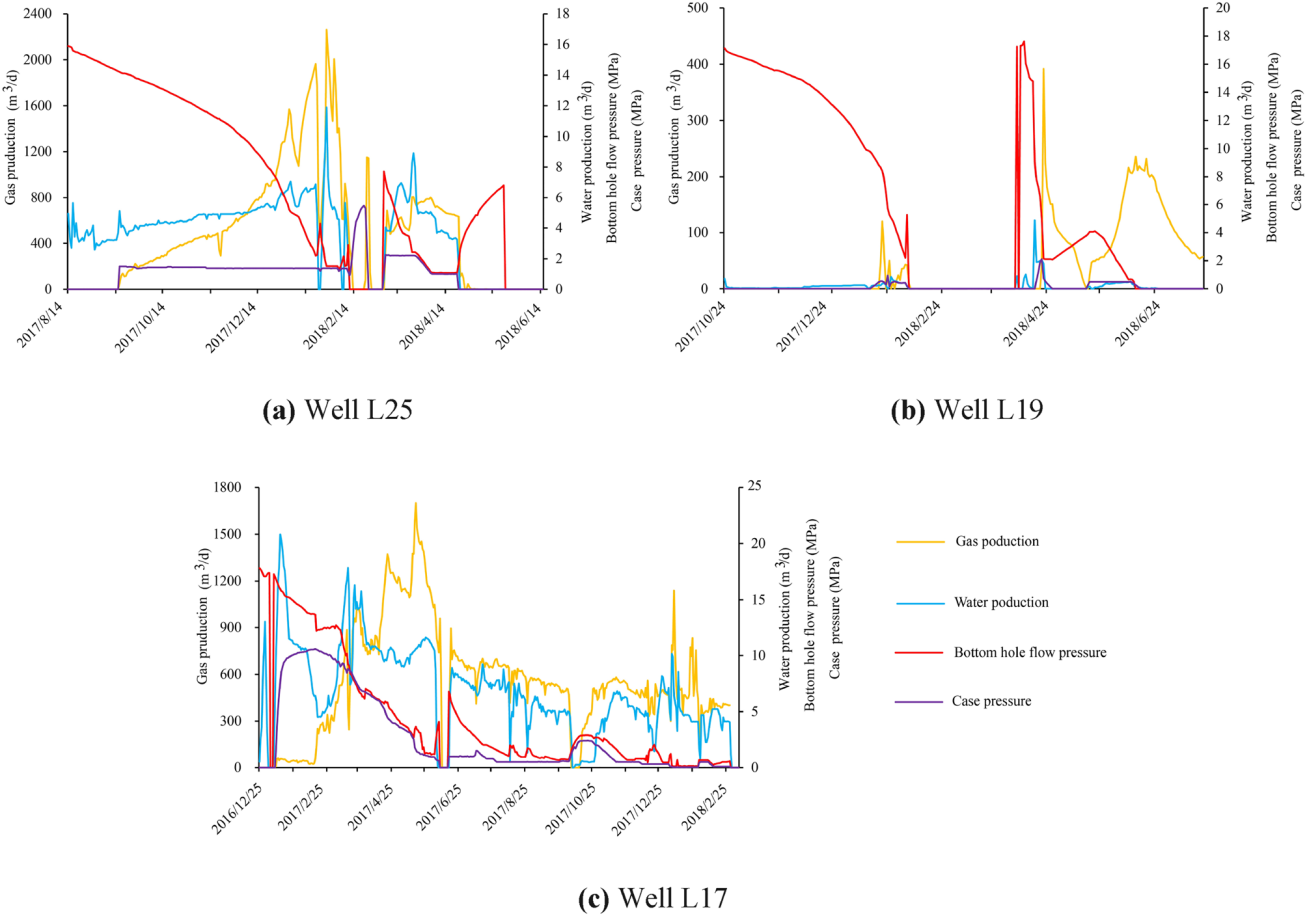
Production characteristics of CBM Wells.

According to the available data, there are four CBM wells (L17, L19, L23, andL25) in the research area, all of which are located in the northern area, with the mining layer being No. 8 + 9 coal seam (Fig. 16b). There is no CBM production in well L23 within half a year, while the production curves of other three wells are shown in Fig. 17. The gas production of well L25 is 0–2262 m^3^/d (avg. 469 m^3^/d). The gas production of well L19 is 0–392 m^3^/d (avg. 41 m^3^/d). The gas production of well L17 is 0–1700 m^3^/d (avg. 431 m^3^/d).

The comprehensive recoverability evaluation score of well L17, L19, L23 and L25 are 0.49, 0.43, 0.50 and 0.51, respectively, which belong to the Class IV areas and have poor production. However, the main factor restricting the efficient production in these four wells is their inadequate development conditions. Factors such as extremely low permeability and stress-induced compression due to negative microstructure restrict the extension of hydraulic fractures (Table 5). As a result, the reservoir transformation is compromised, which limits the release of CBM resources. Given these observations, it can be inferred that the deep CBM recoverability evaluation system demonstrates good applicability in assessing the production potential of these wells.

**Table 5:** The development well parameters in the research area.

Well	Permeability /mD	Elastic modulus/GPa	Stress zoning	Horizontal stress difference	Structural characteristics
L17	0.13	26.18	29.68	9.39	Negative microstructure
L19	0.05	22.40	37.03	11.16	Negative microstructure
L23	0.01	18.19	27.91	6.81	Negative microstructure
L25	0.15	13.4	32.43	8.81	Negative microstructure

## Conclusions

Using the multi-level fuzzy quantitative evaluation method, the deep CBM recoverable favorable area in Linxing Block can be evaluated and classified. The main conclusions are as follows:

1. The research area has two main mineable coal seams, No.4 + 5 and No.8 + 9. The thickness of No.4 + 5 coal seam is between 0.7 and 6.68 m, while thickness of No.8 + 9 coal seam ranges from 2 to 15.43 m. The gas content of No.4 + 5 coal seam in the study area is 0.41–16.39 m^3^/t (avg. 8.14 m^3^/t), the gas content of No.8 + 9 coal seam is 3.64–32.76 m^3^/t (avg. 13.76 m^3^/t). The coal seams are well-preserved and surrounded by mudstone, sandy mudstone, and sandstone. Groundwater salinity suggests a stagnant water environment that supports CBM preservation. Therefore, deep CBM in the research area shows high gas content and oversaturation geological characteristics.
2. The research area has complex in-situ stress conditions, indicating a high-stress environment. The S_hmin_ of No.4 + 5 coal seam is 11.33–35.49 MPa (avg.26.09 MPa), the S_Hmax_ No.4 + 5 coal seam is 13.57–51.84 MPa (avg.33.01 MPa). The S_hmin_ of No.8 + 9 coal seam is 11.98–38.99 MPa (avg.27.79 MPa), the S_Hmax_ No.8 + 9 coal seam is 14.23–53.55 MPa (avg.34.77 MPa), which demands higher requirements for deep coal reservoir transformation techniques. However, in the northern region, the positive microstructure and gentle microstructure are more developed, and the in-situ stress is small, which is conducive to the development of CBM.
3. The comprehensive analysis shows that the comprehensive score of No.4 + 5 coal seam recoverable favorable area varies from 0.36 to 0.81 (avg. 0.56), while the No.4 + 5 coal seam is 0.31 to 0.91 (avg. 0.63). The Level II area is the primary area for the development of No.4 + 5 coal seam, which is distributed in the northeast of the research area. The Level I area and Level II area of No.8 + 9 coal seam is located in the northeast of the research area, with little difficulty in reservoir transformation and high production potential. The Level III area is used as the undertaking area of production of deep CBM and has better exploration prospects. The Level IV area has a large distribution range, but the resource conditions and development conditions are poor, which is not conducive to CBM exploitation. Further analysis reveals that the resource conditions within the Level I and Level II favorable area are generally superior to the development conditions, and these favorable areas are classified as Class A, which is located in the northeast of the research area. The reservoir transformation is relatively difficult and prone to the characteristics of CBM enrichment but not high production (Supplementary information 1).

### Supplementary Information

Supplementary Information.

## Data Availability

The data that support the findings of this study are available from China National Offshore Oil Corporation but restrictions apply to the availability of these data, which were used under license for the current study, and so are not publicly available. Data are however available from the authors upon reasonable request and with permission of China National Offshore Oil Corporation. If you want to request the data from this study, please contact the author B.C. (E-mail address: boboaq410323@163.com).
