# Supplementary material for: Evaluation of recoverable potential of deep coalbed methane in the Linxing Block, Eastern Margin of the Ordos Basin
Source: Sci Rep. 2024 Apr 22;14:9192. doi: 10.1038/s41598-024-59128-x (PMC11035572; doi:10.1038/s41598-024-59128-x)
Supplement: Supplementary file 1 — Supplementary Information. [file 41598_2024_59128_MOESM1_ESM.docx]

Supplementary

The datasets generated and analysed during the current study are not publicly available because we have signed a confidentiality agreement with China National Offshore Oil Corporation (they provided the experimental data in this manuscript). These data can only be used for paper publication, and we cannot share the raw data, but they can be cited from the paper.
